# The metabolic cost of resistance and hypervirulence in Klebsiella pneumoniae: A source of novel therapeutic targets

**DOI:** 10.17179/excli2026-9698

**Published:** 2026-09-29

**Authors:** Eloia Emanuelly Dias Silva, Roberto Vivas da Silveira, Wallison Oliveira Vieira, Ronaldy Santana Santos, Lucas Alves da Mota Santana, Rajiv Gandhi Gopalsamy, Adriana Gibara Guimarães, Sona Arun Jain, Lysandro Pinto Borges

**Affiliations:** 1 Federal University of Sergipe, Northeast Biotechnology Network (RENORBIO), Av. Marcelo Déda Chagas, São Cristóvão, 49000-100 Sergipe, SE, Brazil; 2 Federal University of Sergipe, Graduate Program in Parasitic Biology, Av. Marcelo Déda Chagas, São Cristóvão, 49000-100 Sergipe, SE, Brazil; 3 University of São Paulo, Department of Clinical Analysis and Toxicology, Av. Prof. Lineu Prestes, São Paulo, 05508-900, Brazil; 4 Federal University of Sergipe, Graduate Program in Dentistry, R. Cláudio Batista, Aracaju, 49060-108 Sergipe, SE, Brazil; 5 Division of Phytochemistry and Drug Design, Department of Biosciences, Rajagiri College of Social Sciences, Kochi 683104, Kerala, India; 6 Federal University of Sergipe, Department of Pharmacy, Av. Marcelo Déda Chagas, São Cristóvão, 49000-100 Sergipe, SE, Brazil; 7 Federal University of Sergipe, Department of Morphology, Av. Marcelo Déda Chagas, São Cristóvão, 49000-100 Sergipe, SE, Brazil

## ⁯⁯

The emergence of hypervirulent carbapenem-resistant* Klebsiella pneumoniae* (hv-CRKP) represents one of the most concerning evolutionary convergences in contemporary clinical microbiology. This strain, first reported in Chinese hospitals in 2018, results from plasmid transfer between hypervirulent strains (hvKP) and carbapenemase-producing strains (CRKP), evolutionarily converging into hv-CRKP, a highly pathogenic and resistant strain (Gu et al., 2018[3]; Wang et al., 2025[10]). Carbapenem resistance is driven by the presence of carbapenemase genes and may be complemented by reduced expression of or mutations in porin genes and by overexpression of the *AcrAB-TolC* efflux pump system. Hypervirulence, in turn, is conferred by loci carried on virulence plasmids or mobile integrative elements, particularly genes regulating the polysaccharide capsule and the hypermucoviscous phenotype (*rmpA*, *rmpA2*, and *magA*), as well as siderophore encoding operons involved in iron acquisition (Wang et al., 2025[10]).

The acquisition of multidrug resistance plasmids, combined with the maintenance of a thick capsule and other virulence attributes, should impose a considerable energetic burden on the bacterial cell. According to the classical fitness cost model, this investment would reduce growth rate, competitive ability, or lineage stability in the absence of selective pressure (Vanacker et al., 2023[9]). Fitness cost refers to the relative reduction in an organism's ability, in this case, a resistant bacterium, to survive, grow, compete, reproduce, and transmit its genes within a given environment compared with a fitter reference genotype (Vanacker et al., 2023[9]). In bacteria, this cost arises when plasmids and other genetic elements acquired through horizontal transfer impose additional demands associated with the replication, maintenance, and expression of exogenous DNA (Pal and Andersson, 2024[6]).

In the context of multidrug resistance, fitness costs are observed when resistance mechanisms exert pleiotropic effects on global regulators, simultaneously affecting energy metabolism, stress responses, virulence, and cellular homeostasis (Pal and Andersson, 2024[6]). However, successful hv-CRKP clones appear to circumvent these costs while preserving antimicrobial resistance, hypervirulence, and a high capacity for dissemination (Huang et al., 2026[5]). This scenario suggests that the convergence of resistance and virulence depends not only on the acquisition of new genes but also on physiological mechanisms capable of compensating for the costs of their expression.

RNA-seq-based transcriptomic studies have expanded our understanding of hv-CRKP, but most analyses remain focused on the most predictable candidate genes. Carbapenemase genes, such as *blaKPC*, and classical hypervirulence markers, such as *rmpA* and *iucA*, are frequently assessed to determine whether they are differentially expressed (Hao et al., 2022[4]). Demonstrating the overexpression of resistance or virulence determinants confirms that these systems are active (Dai et al., 2025[2]). However, as exemplified by hv-CRKP, these studies do not necessarily explain how the bacterium energetically sustains their simultaneous operation. Consequently, there is a risk of describing the visible components of the phenotype while overlooking the metabolic infrastructure that enables their maintenance.

Alterations in the tricarboxylic acid cycle, amino acid metabolism, bacterial respiration, and systems for the uptake and utilization of alternative carbon sources may reveal how these lineages reorganize the flow of energy and biosynthetic precursors (Yao et al., 2025[11]). Rather than regarding such changes as secondary effects, they may be investigated as central components of adaptation. The production of capsules, siderophores, efflux pumps, and carbapenemase enzymes requires resources that must be acquired, conserved, or redistributed. Thus, integrating differentially expressed genes analysis, metabolic pathway enrichment, and metabolic flux assessment may clarify how hv-CRKP simultaneously sustains its resistance and virulence without incurring a fitness loss incompatible with its epidemiological expansion.

The identification of these metabolic transcriptomic signatures also has direct therapeutic implications (Sahu et al., 2026[8]). Pathways that compensate for the energetic costs of resistance and hypervirulence may constitute less apparent yet potentially exploitable vulnerabilities. The study by Ren et al. (2026[7]) suggests that the pathogenicity and survival of *K. pneumoniae* depend on the coupling between metabolism and virulence, whereby metabolic pathway reprogramming compensates for tissue-associated fitness costs and enables adaptation to host-related stresses. Similarly, Curtsinger et al. (2025[1]) argue that the acquisition of resistance mechanisms imposes a metabolic burden that forces bacteria to reorganize their biosynthetic pathways, thereby generating exploitable collateral sensitivities.

In light of the above, efforts to control hv-CRKP should not be limited to the development of new compounds targeting the cell wall or carbapenemases. Strategies capable of disrupting energy production, nutrient acquisition, or the carbon redistribution required to maintain the convergent phenotype may also be considered. Blocking this metabolic compensation could destabilize the bacterium's physiological balance, restore previously neutralized evolutionary costs, and increase its susceptibility to host defenses and antimicrobial agents. In this context, hv-CRKP RNA-seq datasets must be reexamined not only to identify the genes that make this bacterium resistant and virulent but also to uncover the metabolic pathways that enable it to bear the cost of both.

## Notes

Eloia Emanuelly Dias Silva and Lysandro Pinto Borges (Federal University of Sergipe, Northeast Biotechnology Network (RENORBIO), Av. Marcelo Déda Chagas, São Cristóvão, 49000-100 Sergipe, SE, Brazil; E-mail: lysandro.borges@gmail.com) contributed equally as corresponding author.

## Declaration

### Author contributions

EEDS conceived the study and reviewed the scientific literature. EEDS, RVS and WOV prepared the first draft. RSS, LAMS, AGG, RGG, SAJ and LPB reviewed the paper. All authors contributed to revisions of the paper and approved the final version.

### Funding and assistance

This research is being conducted with support from the Brazilian Agency for the Support and Evaluation of Graduate Education (CAPES). Funding Code 001 (EEDS: 88887.205235/2025-00; RSS: 88887.082794/2024-00)

### Conflict of interest

The authors have no conflict of interest.

### Artificial Intelligence (AI) - assisted technology

No generative AI tools were used.
